# Using experiential learning and authentic assessments to support students to become competent health promotion practitioners

**DOI:** 10.1002/hpja.654

**Published:** 2022-09-06

**Authors:** Karen Anderson, Sabrina Gupta, Fernanda Nava Buenfil, Glenda Verrinder

**Affiliations:** ^1^ Department of Community and Allied Health, Violet Vines Centre for Rural Health Research La Trobe Rural Health School Bendigo Vic. Australia; ^2^ School of Psychology and Public Health, College of Science, Health and Engineering La Trobe University Melbourne Vic. Australia; ^3^ Department of Community and Allied Health La Trobe Rural Health School Bendigo Vic. Australia

**Keywords:** curriculum, educational techniques, health promotion, health promotion competencies, higher education, needs assessment, pedagogy

## Abstract

**Title:**

Using experiential learning and authentic assessments to support students to become competent health promotion practitioners.

**Issue Addressed:**

The aim of this article is to describe how experiential learning, authentic assessments, community development and ethical principles were consolidated in the design and delivery of a health promotion planning and evaluation subject (HPE) during 2019 to 2021. Experiential learning and authentic assessments were used to guide the development of health promotion competencies in line with the International Union for Health Promotion and Education (IUHPE) Core Competencies and Professional Standards for Health Promotion. Students were required to complete three sequential authentic assessments. Ethics approval was granted for students to undertake a needs/assets assessment with a local community group following which, students completed a literature review and planned a community development program.

**Methods:**

The subject comprises 10 h of weekly engagement over a 12‐week semester with weekly topics following a program logic model. Working in teams and individually, students work with a local community group to assess their needs/assets and establish priority areas regarding health and wellbeing. This then informed the development of a health promotion program and evaluation plan. Students undertook three sequential authentic assessment tasks: (i) needs/assets report, (ii) a literature review and (iii) a program folio. Retrospective anonymous student feedback on subject (SFS) data from 2019 to 2021 was used to evaluate HPE.

**Results and Discussion:**

HPE provides students with the opportunity to understand ethical principles and processes, engage with stakeholders in the community and develop qualitative research skills, to plan and evaluate health promotion programs. SFS scores have improved with an overall score of 3.7 (response rate 49.44%) in 2019 to 4.3 (response rate 39.58%) in 2020 and 4.04 (response rate 28.57%) in 2021.

**Conclusion:**

Students acquire a broad range of knowledge and skills in line with IUHPE Core Competency and Professional Standards for Health Promotion preparing them for their future professional practice.

**So what?:**

These teaching and learning experiences show that students can develop health promotion competencies through sound pedagogical approaches, both online and face‐to‐face and in challenging environments.

## 1 INTRODUCTION

Competency standards in health promotion offer a basis for education and training, and for strengthening the health promotion workforce by setting the standards for health promotion work.^1^, ^2^, ^3^ Embedding competencies such as the International Union for Health Promotion and Education (IUHPE) Core Competencies and Professional Standards for Health Promotion^4^ in the design and delivery of the curricula, ensures students are taught the necessary knowledge and skills to develop, implement and evaluate health promotion policies and practices. This then contributes towards building a competent health promotion workforce.^1^

Developing competence requires the coordination and integration of knowledge, skills, competencies and values.^5^ One approach to developing competence is through experiential learning. Experiential learning is a pedagogical approach that uses life experiences whereby the learner is directly in touch with the realities being studied.^6^ It places students at the centre of learning and occurs when certain activities (created by teachers) are carried out. As a result of these activities, a range of skills and competencies are developed.^5^, ^6^

Authentic assessments are one experiential learning approach that can be used to develop competence. An authentic assessment is one that requires the application of what students have learned to a new situation and provides students with more freedom to demonstrate their competence.^7^ According to Wiggins,^8^ an assignment is authentic if it is realistic, requires judgement and innovation, asks students to “do” the subject, and replicates or simulates the contexts in which adults are “tested” in the workplace. It assesses students' ability to use a repertoire of knowledge and skills and allows appropriate opportunities to rehearse, practice, consult resources and get feedback on and refine performances and products.

Health Promotion Program and Evaluation (HPE) is a core third‐year subject offered in the Health Promotion major of the Bachelor of Health Sciences at a university located in Victoria, Australia; approximately 50 students a year complete this subject across the metropolitan and regional campuses. In this subject, students examine and apply the health promotion planning and evaluation cycle.^9^, ^10^ The intended learning outcomes (ILOs) of the subject focus on analysis and application of health promotion and community development principles, consideration of ethical practice requirements as well as the impact of wider socio‐ecological factors on health and wellbeing. A series of sequential, authentic assessment tasks^7^ are used to step students through the program planning cycle and to enable them to meet the ILOs and the IUHPE Core Competencies and Professional Standards for Health Promotion.^4^

In HPE, we take the approach of consolidating health promotion competencies using the pedagogical approaches outlined above.^6^, ^10^, ^11^ In addition, principles and practices of research ethics and community development are an integral part of the HPE curricula.^12^ This has been achieved through the inclusion of research ethics and training materials within the curricula that also align with community development. Community development is a process of “working with people as they define their own goals, mobilise resources and develop action plans for addressing problems they collectively have identified.”^13^ Community development is a holistic approach grounded in principles of empowerment, human rights, inclusion, social justice, self‐determination and collective action.^14^ These principles are embedded within the HPE curricula.

In this article, we describe how experiential learning, authentic assessments, community development and ethical principles were consolidated in the design and delivery of HPE to develop competent health promotion graduates and report on anonymous student feedback over the period of 2019 to 2021.

## 2 METHOD

### 2.1 Subject overview

**FIGURE 1:**
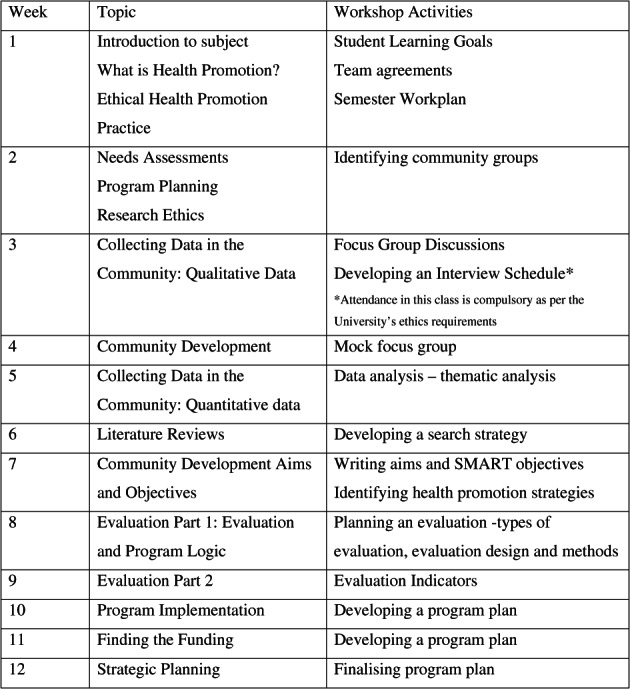
Subject calendar

The subject comprises 10 h of weekly engagement over a 12‐week semester: 1 h of pre‐workshop learning via the subject learning management system (LMS); a 2‐h workshop; and 7 h per week of self‐directed learning tasks. Prior to the COVID‐19 pandemic, lectures and workshops were delivered face‐to‐face. However, in 2020, delivery moved to online mode and continued in 2021 due to Victoria's ongoing public health lockdowns. The topics covered follow a program‐logic model^15^, ^16^ and include needs/assets assessment, collecting data in the community, ethical and community development principles, program planning including developing aims and objectives, implementation, evaluation and program logic, strategic planning, and funding, Figure 1 shows the weekly topics for this subject.

In week one of the subject, students were provided with a scenario that established the context for the subject and assessment. Working in small teams (3‐5 students) students were asked to work with a local community to assess the needs/assets of that community and establish community priority areas regarding health and wellbeing with the aim of developing a health promotion program and evaluation plan.

Human Ethics Committee (HEC) permission for students to conduct focus groups to carry out the needs/assets assessment, has been received from the University's Human Ethics Sub‐committee (S17007).

### 2.2 Assessments

Students individually and in teams developed a health promotion program based on a community development approach. To do this, students undertook three authentic assessment tasks: (i) needs/assets report, (ii) a literature review and (iii) a program folio. Table 1 demonstrates the link between the program planning cycle, assessments, and health promotion competencies.

**TABLE 1:** Assessment and competencies

Program planning cycle stage	Assessment	HP competency domain	Competency statements
Assessment (primary data collection)	*Assessment 1. Needs and Assets Assessment Report* In small team's student conduct a focus group with participants from a local community, who represent a subgroup of the population of the community. Students are required to: Develop questions to be asked of their community members during the semi‐structured focus group interview Conduct a focus group interview with 6‐8 people from the local community (30 min to 1 h in duration) Record the interview, take notes and observe during it Transcribe the recording. Conduct a thematic analysis of the transcript Write a needs/assets assessment report to be submitted for assessment.	Enable Change	1.2 Use Health Promotion approaches which support empowerment, participation, partnership and equity to create environments and settings which promote health 1.3 Use community development approaches to strengthen community participation and ownership and build capacity for Health Promotion action
		Advocate for Health	2.5 Facilitate communities and groups to articulate their needs and advocate for the resources and capacities required for Health Promotion action
		Communication	4.1 Use effective communication skills including written, verbal, nonverbal, listening skills and information technology 4.2 Use electronic and other media to receive and disseminate Health Promotion information 4.3 Use culturally appropriate communication methods and techniques for specific groups and settings 4.4 Use interpersonal communication and groupwork skills to facilitate individuals, groups, communities, and organisations to improve health and reduce health inequities
		Leadership	5.2 Use leadership skills which facilitate empowerment and participation (including teamwork, negotiation, motivation, conflict resolution, decision‐making, facilitation and problem‐solving) 5.3 Network with and motivate stakeholders in leading change to improve health and reduce inequities 5.4 Incorporate new knowledge and ideas to improve practice and respond to emerging challenges in Health Promotion 5.5 Contribute to mobilising and managing resources for Health Promotion action 5.6 Contribute to team and organisational learning to advance Health Promotion action
		Assessment	6.1 Use participatory methods to engage stakeholders in the assessment process 6.2 Use a variety of assessment methods including quantitative and qualitative research methods 6.5 Identify the health needs, existing assets, and resources relevant to Health Promotion action 6.7 Identify priorities for Health Promotion action in partnership with stakeholders based on best available evidence and ethical values
Assessment (secondary data collection)	*Assessment 2. Literature Review* Each student is to review literature that is relevant to EITHER a specific theme that emerged from the needs assessment, OR programs/practices that have been implemented and evaluated to address a health need/theme, OR policies, relating to the community need/asset assessment conducted for assessment one. Students are required to: Develop relevant and answerable practice‐related question. Develop strategies for searching in databases for evidence that is relevant to each of the practice‐related questions. Search databases to acquire papers that contain relevant evidence. Select, from the search yield, papers to include in the literature review. Critically appraise each of the papers selected for inclusion in the review.	Communication	4.1 Use effective communication skills including written, verbal, nonverbal, listening skills and information technology 4.2 Use electronic and other media to receive and disseminate Health Promotion information
		Assessment	6.3 Collect, review, and appraise relevant data, information, and literature to inform Health Promotion action 6.5 Identify the health needs, existing assets, and resources relevant to Health Promotion action 6.7 Identify priorities for Health Promotion action in partnership with stakeholders based on best available evidence and ethical values
Planning, Implementation and Evaluation	*Assessment 3. Program Plan and Evaluation* The third assignment for the subject involves developing a health promotion program based on a community development approach. The program is to be designed and developed as a response to the community needs and assets which were identified by community members in the focus groups conducted for the team's needs/assets assessment project (primary data) and informed by your literature reviews (secondary data). Students are required to: Provide a rationale for the program. Use the program logic model to develop a program including goal, objectives, strategies, stakeholder identification, resources. Evaluation plan including evaluation design, data collection and analysis procedures, dissemination plan.	Communication	4.1 Use effective communication skills including written, verbal, nonverbal, listening skills and information technology 4.2 Use electronic and other media to receive and disseminate Health Promotion information 4.3 Use culturally appropriate communication methods and techniques for specific groups and settings 4.4 Use interpersonal communication and groupwork skills to facilitate individuals, groups, communities and organisations to improve health and reduce health inequities
		Planning	7.2 Use current models and systematic approaches for planning Health Promotion action 7.3 Develop a feasible action plan within resource constraints and with reference to existing needs and assets 7.4 Develop and communicate appropriate, realistic, and measurable goals and objectives for Health Promotion action 7.5 Identify appropriate Health Promotion strategies to achieve agreed goals and objectives
		Implementation	8.1 Use ethical, empowering, culturally appropriate, and participatory processes to implement Health Promotion action
		Evaluation and Research	9.1 Identify and use appropriate Health Promotion evaluation tools and research methods 9.2 Integrate evaluation into the planning and implementation of all Health Promotion action 9.3 Use evaluation findings to refine and improve Health Promotion action 9.4 Use research and evidence‐based strategies to inform practice 9.5 Contribute to the development and dissemination of Health Promotion evaluation and research processes

In assessment one, students worked in team to identify a local community group and conduct a focus group to ascertain the needs and assets of the group. Students were required to draw on their existing social networks to recruit a local group who met regularly and who did not meet for a disease‐related reason. Some examples of groups that students could conduct a focus group with included sporting and church groups, parents and volunteer groups, book clubs and choirs. Students were required to demonstrate ethical practice by following the ethical requirements approved by the HEC (privacy and confidentiality, informed consent). Students then developed a semi‐structured interview schedule. Examples of questions included “What factors or issues make it easier or more difficult for you to achieve good social well‐being?”, “What, if anything, would you like to see changed in your community to help you live health and happy life?”. They then conducted a focus group of up to 1 h, recorded, scribed, and transcribed the discussion and undertook a thematic analysis of the transcript before writing a needs assessment report. Student learning was supported via the content embedded in LMS, as well as through the participation in workshop activities such as a mock focus group and practicing coding.

In assessment two, students undertook secondary data collection by means of a narrative literature review, as part of the assessment stage of the program planning cycle. In this assessment, students worked with their team to identify specific themes that emerged from their needs/assets assessment, or programs/practice, or policies related to those themes. Whilst students worked together to identify themes, they carried out the literature review independently on agreed needs/assets or programs/practice, or policies. The literature review enabled students to understand the importance of evidence‐informed health promotion practice. In addition, this assessment was a hurdle, meaning, students must achieve a pass grade on this task to have successfully completed the subject.

The program folio was the final part of the sequence of assessments (see Table 1) where students worked in their team to bring together assessments one and two to develop a program plan. Students gained skills in developing a rationale for their program and then used the program‐logic model^15^, ^16^, ^17^ to write a goal, SMART objectives and identify suitable strategies and relevant stakeholders. Students also acquired skills to develop an evaluation plan for their program. This included identifying appropriate evaluation methods to conduct impact, process and outcome evaluations. Students gained experience in identifying evaluation questions and indicators, identifying data collection and analyses procedures. In addition, students considered resources required for evaluation, and methods for the dissemination of the evaluation findings. Students also demonstrated community development principles and processes such as decision‐making, empowerment and mobilisation of resources.^14^ This assessment and the program logical model template (provided to students to complete as part of the assessment) was based on the Victorian Government's Integrated Health Promotion Plan.^18^

### 2.3 Subject evaluation

HPE is formally evaluated each year by students using the University's “Student Feedback on Subjects” (SFS) evaluation survey. The aim of the SFS is to improve and maintain student experience and subject quality. The SFS survey consists of eight 5‐point scale items (eg, The learning outcomes of the subject were clear to me, I received constructive feedback on my work, Overall, I was satisfied with the quality of this subject) and three qualitative items which included: What were the best aspects of this subject? What aspects of this subject were most in need of improvement? The SFS is administered and analysed centrally by the University's survey team to maintain student anonymity. A SFS report is sent to the subject coordinator once final subject results have been released to students. The data is presented as 3‐year quantitative trend analysis shown as a mean score and raw qualitative comments. Ethics approval to use retrospective anonymous student data from 2019 to 2021 was received from the University's Human Research Ethics Committee (No. HEC21426).

## 3 RESULTS AND DISCUSSION

Students in HPE acquired a broad range of knowledge and skills to prepare them for their future professional practice in health promotion. Health promotion competencies are at the core of HPE. The attainment of competencies was supported using sequential, authentic assessments, and informed by ethical practice and community development principles. Based on reflections of our teaching practice and the SFS data, a pedagogical framework for HPE was developed to provide teaching staff and students with a clear plan for achieving the subject ILOs, something that is essential when implementing authentic assessments which in turn contributes to students' learning and professional development.^1^, ^19^ Through sound pedagogical approaches, and using a combination of teaching and learning experiences, student learning outcomes aligned with the IUHPE Core Competencies and Professional Standards for Health Promotion.

### 3.1 Lessons from strategies: Feedback and subject performance

HPE has been delivered in a variety of modes: face‐to‐face, blended (a combination of face‐to‐face and online) and fully online. Despite changes to the delivery mode, the use of authentic, sequential assessments, aligned to the program‐logic model^16^, ^17^ has been maintained to enable students to develop the knowledge and skills required meet the IUHPE Core Competencies and Professional Standards for Health Promotion (see Table 1).

The use of authentic assessments in the subject has provided students with the opportunity to understand ethical principles and processes, engage with stakeholders in the community and develop qualitative research skills such as developing a data collection tool, conducting a focus group, transcribing, data analyses, interpretation, and dissemination of findings. Qualitative feedback from students showed they like the freedom to choose their community group and enjoy engaging with the community:

*Freedom to choose our own focus group (for our location and learning benefit)* (Student 2019).

*Actually, engaging with the community in the needs assessment was really fun and interesting* (Student 2019).

Despite the challenges that came with the COVID‐19 pandemic restrictions, the use of authentic assessment was maintained. For example, in 2020, with only 2 weeks to transition to an online mode due to the COVID‐19 related lockdowns, students were unable to conduct a focus group. Instead, students participated in mock focus groups with their workshop peers and were then provided with an audio‐recording of focus groups from the previous year so that students could still undertake transcription and analyses. However, students expressed their disappointment at not being able to participate in a real focus group:

*Not being able to conduct the focus interview was very disappointing but using previous years audio recordings was difficult. It may have been better to conduct one with our peers online*. (Student 2020)

Reflecting on this feedback in 2021, ethics approval was sought for students to be able to conduct their focus group online. This provided students the opportunity to experience and develop the skills required to work remotely. With each experience, students have been required to use their judgement, be innovative and draw on their repertoire of knowledge and skills to negotiate a complex task in line with important elements of authentic assessments.^8^, ^19^ As this student said:

*This subject enabled me to gain experience and practice in completing reports, conducting focus groups, and communicating with the community in a similar way to a professional health role*. (Student 2021).

Authentic assessments were designed to equip students with specific knowledge and skills (competencies) relevant to real world settings.^7^, ^19^ The qualitative feedback from students consistently showed that students in HPE could see how the sequencing of the subject works and were able to see the links to their future professional practice as can be noted in the following student comments:

*This subject is great for getting some experience about how to do some real‐world work. It has been more practical, and all relates to the same thing. Our learning also depends on what we have organised and done!* (Student 2019).

*The subject is very relevant to real life and gives us the skills to be competent in future workplaces* (Student 2020).

*Collectively, the assessments were interesting to undertake. They all related to each other, so it felt as though we truly were investigating and health promoting for our particular focus groups*. (Student 2021).

The use of experiential learning, combined with authentic assessments stepped students through the program planning cycle and enabled students to develop the knowledge and skills required to meet the IUHPE Core Competencies and Professional Standards for Health Promotion^4^ and prepared them for their future practice as health promotion practitioners. The use of experiential learning and authentic assessments in subjects like HPE can aid in motivating and inspiring students to examine dimensions of health promotion practice that might otherwise be overlooked in more traditional teaching approaches. It encourages social aspects of learning by enabling active participation and deeper learning. The use of authentic assessments such as the needs and assets assessment and program folio in HPE provided students the opportunity to learn through experience and engagement with their peers and the wider community whilst considering real world public health issues.^7^, ^19^ The impact of the different pedagogical approaches implemented through the subject can also be noted from the formal university‐driven student feedback on the subject. SFS quantitative scores improved during the pandemic from an overall score of 3.7 (response rate 49.44%) in 2019 to 4.3 (response rate 39.58%) in 2020 and 4.04 (response rate 28.57%) in 2021. Importantly, the impact of the subject on student learning has been sustained over time.

### 3.2 New opportunities for delivering HPE

Students gained knowledge and skills relevant to all aspects of the program planning cycle particularly in assessment three whereby they were able to plan the implementation of their program in either face‐to‐face and/or online contexts. Using online technologies like Zoom and MS Teams to facilitate group meetings and share documents, develop written and verbal communication skills, teamwork, and leadership skills, created opportunities for student conversations around digital health promotion including the challenges and the barriers. In future, these digital technology skills will be a required health promotion competency.^20^, ^21^ Opportunities for digital engagement were also presented to the teaching team for future iterations of the subject.

In 2022, HPE is being delivered online and face‐to‐face simultaneously (hybrid‐mode), thus providing students with greater flexibility and choice about how they participate in the subject. This approach will be evaluated through the formal university‐driven student feedback on the subject process. In addition, a formal peer review and observations of teaching will be undertaken. Changes to the delivery of the subject will be considered once this process has been completed.

## 4 STRENGTHS AND LIMITATIONS

HPE is well structured, supported with theory and the learning outcomes are well aligned with the IUHPE Core Competencies and Professional Standards for Health Promotion. This combined with the use of an experiential learning approach provides students with practical knowledge and skills required for their future practice as health promotion practitioners.

Despite SFS informing strategies subject improvement there are some limitations. For example, it has been argued that SFS is conducted to serve the needs of university and regulatory bodies to meet notions of what quality teaching may look like.^22^ Other authors have suggested the use of informal feedback throughout the subject has been more effective in implementing strategies to improve the quality of subjects in a timely manner.^11^ Another potential limitation of the SFS is student bias. SFS results and response rates can be influenced by personality, age, gender, and physical appearance,^23^, ^24^ delivery mode preference and survey fatigue.^25^ For example, it is unknown if the dip in the overall score for HPE in 2021, was due to some of the above reasons, the change in mode of delivery and/or the impact of the pandemic. Future iterations of HPE will need to consider informal ways of gathering student feedback to compliment SFS results.

## 5 CONCLUSION

Three years of student feedback data shows that HPE is an undergraduate subject that creates opportunity for students to develop health promotion competencies. HPE has been delivered in a variety of modes with each one providing opportunities for flexible learning and the development of skills which will be required for future health promotion practice. The subject uses experiential learning and sequential, authentic assessment to enable students to apply health promotion theory to plan, implement and evaluate a health promotion program to meet the IUHPE Core Competency and Professional Standards for Health Promotion. HPE equips students to be skilled, ethical, and principled health promotion practitioners who are adaptable and ready to address unprecedented public health challenges.

## AUTHOR CONTRIBUTIONS

Karen Anderson, Sabrina Gupta and Fernanda Nava Buenfil contributed to the conceptualisation and preparation of the manuscript. All authors contributed to the development of the subject in focus in this manuscript and were involved in revising the manuscript for important intellectual content.

## CONFLICT OF INTEREST

The authors declare there are no conflicts of interest.

## ETHICS APPROVAL

This project has approval from the La Trobe University College of SHE Human Ethics sub‐committee (HEC21426).
